# Fermentable fiber-induced hepatocellular carcinoma in mice recapitulates gene signatures found in human liver cancer

**DOI:** 10.1371/journal.pone.0234726

**Published:** 2020-06-19

**Authors:** Darshan Shimoga Chandrashekar, Rachel M. Golonka, Beng San Yeoh, David J. Gonzalez, Mathias Heikenwälder, Andrew T. Gerwirtz, Sooryanarayana Varambally, Matam Vijay-Kumar

**Affiliations:** 1 Department of Pathology, University of Alabama at Birmingham, Birmingham, AL, United States of America; 2 Department of Physiology and Pharmacology, University of Toledo College of Medicine and Life Sciences, Toledo, OH, United States of America; 3 Department of Pharmacology, School of Medicine, and The School of Pharmacy and Pharmaceutical Sciences, University of California, San Diego, La Jolla, CA, United States of America; 4 Division of Chronic Inflammation and Cancer, German Cancer Research Center (DKFZ), Heidelberg, Germany; 5 Center for Inflammation, Immunity and Infection, Institute for Biomedical Sciences, Georgia State University, Atlanta, GA, United States of America; 6 Comprehensive Cancer Center, University of Alabama at Birmingham, Birmingham, AL, United States of America; 7 Department of Medical Microbiology and Immunology, University of Toledo College of Medicine and Life Sciences, Toledo, OH, United States of America; University of Navarra School of Medicine and Center for Applied Medical Research (CIMA), SPAIN

## Abstract

Hepatocellular carcinoma (HCC), the most malignant form of primary liver cancer, is the fourth most prevalent cause of cancer mortality globally. It was recently discovered that the dietary fermentable fiber, inulin, can reprogram the murine liver to favor HCC development in a gut microbiota-dependent manner. Determining the molecular pathways that are either over expressed or repressed during inulin-induced HCC would provide a platform of potential therapeutic targets. In the present study, we have combined analysis of the novel inulin-induced HCC murine model and human HCC samples to identify differentially expressed genes (DEGs) in hepatocarcinogenesis. Hepatic transcriptome profiling revealed that there were 674 DEGs in HCC mice compared to mice safeguarded from HCC. Kyoto Encyclopedia of Genes and Genomes (KEGG) pathway analysis uncovered enrichment in ECM-receptor interaction, steroid hormone biosynthesis, PPAR signaling pathway, focal adhesion and protein digestion and absorption during inulin-induced HCC. Tandem mass tag based quantitative, multiplexed proteomic analysis delineated 57 differentially expressed proteins, where the over-expressed proteins were associated with cell adhesion molecules, valine, leucine and isoleucine degradation and ECM-receptor interaction. After obtaining the human orthologs of the mouse genes, we did a comparison analysis to level 3 RNA-seq data found in the Cancer Genome Atlas (TCGA) database, corresponding to human HCC (n = 361) and healthy liver (n = 50) samples. Out of the 549 up-regulated and 68 down-regulated human orthologs identified, 142 genes (137 significantly over-expressed and 5 significantly under-expressed) were associated with human HCC. Using univariate survival analysis, we found 27 over-expressed genes involved in cell-cell adhesion and cell division that were associated with poor HCC patient survival. Overall, the genetic and proteomics signatures highlight potential underlying mechanisms in inulin-induced HCC and support that this murine HCC model is human relevant.

## Introduction

Hepatocellular carcinoma (HCC), the most frequent malignancy of the liver, is the fourth most common cause of cancer mortality worldwide [1]. The high rate of fatalities for HCC is typically attributed to being asymptomatic and highly heterogenous in nature. In addition, there is a lack of standardized, non-invasive early-stage biomarkers for detection screening [2]. Notwithstanding, HCC is a complex disease with a variety of etiological risk factors. In a majority of HCC cases, cirrhosis caused by viral infection is found to be the predominant etiology of HCC [3, 4]. Synergy of viral infections and aflatoxin, a frequent fungal contaminant in food, is another potent inducer of HCC [5]. Not to mention, single nucleotide polymorphisms have been strongly associated with HCC risk [6, 7]. This has made chemical induction, viral infection, and genetic manipulation the front-line approaches to study HCC in a murine model [8–10]. Correspondingly, the laboratory mouse is widely considered a suitable organism for studying human relevant diseases because their gene expression patterns can recapitulate human conditions [11, 12].

In our recently discovered novel HCC murine model, we found that a subset (~40%) of Toll-like receptor 5 deficient (T5KO) mice with gut dysbiosis developed HCC after being fed a diet enriched with the fermentable fiber, inulin, for 6 months [13]. A distinguishing factor between the cancer-free and HCC-prone T5KO mice was the early onset development of cholestasis, as indicated by hyperbilirubinemia and elevated bile acids (*alias* cholemia). This suggests that inulin might be one of the ingredients of plant-derived supplements contributing to the reported adverse effects of jaundice and cholestasis [14]. It must be cautioned that purified/refined dietary fibers such as inulin are widely marketed as beneficial supplements, where the U.S. Food and Drug Administration (FDA) has recently approved the labeling of inulin-enriched foods as health-promoting [15].

The exciting finding of inulin-induced HCC has introduced an unexpected etiological risk factor that has potential translational merits needing to be addressed. In the current study, we sought to determine the molecular pathways that are either over or under expressed during dietary fermentable fiber-induced HCC, which would not only delineate how enhanced gut fermentation activity influences liver pathophysiology but would also provide a platform of potential therapeutic targets. In our study, we performed transcriptomic profiling and proteomic analysis of HCC-prone T5KO mice with high bilirubin (T5KO-HB) and compared their gene/protein signatures to T5KO normal bilirubin (T5KO-NB) and wild-type (WT) mice that do not develop HCC upon inulin containing diet (ICD) supplementation. Alongside, we compared the differentially expressed human orthologous genes to the RNA-seq data found in the Cancer Genome Atlas (TCGA) database, corresponding to human HCC and healthy liver samples. Accordingly, we observed that ICD-induced HCC significantly alters the expression of various genes and proteins involved in extracellular matrix (ECM)-receptor interaction and cell cycle functions. Furthermore, we found that there was an overlap in differentially expressed genes in our mouse model and human HCC, where a fraction of the over-expressed genes was associated with poor HCC patient survival. Overall, the transcriptomic and proteomic signatures highlight potential underlying mechanisms in ICD-induced HCC and support that this HCC murine model is human relevant.

## Materials and methods

### Mice model

Toll-like receptor 5 deficient (T5KO) mice were originally generated by Dr. Shizuo Akira, Japan on C57BL/6 background. T5KO mice were bred with C57BL/6 wild-type (WT) mice in our colony to generate their WT littermates. T5KO and their WT littermates were maintained in specific pathogen–free conditions at The University of Toledo. Mice were housed in cages (n = 5 mice/cage) containing corn cob bedding (Bed-O-Cob, The Andersons Co.) and neslets (Cat # CABFM00088, Ancare). The cages were housed at 23°C and underwent a 12-h light/dark phase cycle. Mice were weaned at 22 days and fed grain-based lab chow (LabDiet 5001) *ad libitum* for one week to acclimatize for solid food and to stabilize the intestinal microbiota. Subsequently, four-week-old male mice were maintained on an inulin-containing diet [ICD; a polyfructosan; Orafti® HP; Source: chicory root; inulin content: 100%; and degree of polymerization: > 23; Beneo (Tienen, Belgium); Cat# D12081401] for 6 months. ICD contained a ratio of 75 g (7.5%) inulin + 25 g (2.5%) cellulose per kg diet; 2.5% cellulose was maintained to sustain roughage of stool. We have previously observed that, after 6 months of ICD feeding, the incidence of liver cancer was ~40% in T5KO male mice [13]. After 5 hours of fasting, mice were euthanized, and liver samples were obtained for further analysis described below. All animal experiments were approved by the University of Toledo Institutional Animal Care and Use Committee (IACUC).

### Hepatic transcriptome via RNA-sequencing

RNA-seq analysis was performed by Arraystar, MD as previously described [13]. In brief, RNA extraction, cDNA synthesis and RNA-seq libraries were conducted as previously described [13]. The prepared RNA-seq libraries were qualified using Agilent 2100 Bioanalyzer and quantified by qPCR absolute quantification method. The sequencing was performed using Illumina Hiseq 4000. Raw sequencing data generated from Illumina HiSeq 4000 that pass the Illumina chastity filter were used for the following analysis. Trimmed reads (trimmed 5’,3’-adaptor bases) were aligned to reference genome. Based on alignment statistical analysis (mapping ratio, rRNA/mtRNA content, fragment sequence bias), we determine whether the results can be used for subsequent data analysis. If so, the expression profiling, differentially expressed genes and differentially expressed transcripts were calculated. The novel genes and transcripts are also predicted. Principal Component Analysis (PCA), Correlation Analysis, Hierarchical Clustering, Gene Ontology (GO), Pathway Analysis, scatterplots and volcano plots were performed for the differentially expressed genes in R or Python environment for statistical computing and graphics. The accession number for the unprocessed transcriptomic sequencing data is PRJEB28449.

### Hepatic proteomics

Liver samples were processed and analyzed by LC-MS2/MS3 for identification and quantitation as previously described [13]. In brief, an Orbitrap Fusion (Thermo Fisher Scientific) with an in-line Easy-nLC 1000 (Thermo Fisher Scientific) was utilized for LC-MS2/MS3 experiments. The Orbitrap Fusion was run in data-dependent mode and a survey scan was collected over 500–1200 m/z at a resolution of 120000 in the Orbitrap. For MS2/MS3 analysis, top speed mode was enabled to select the most abundant ions for analysis in a 5 s cycle. Furthermore, the decision tree option was used with charge state and m/z range as qualifiers. MS3 analysis was conducted using the synchronous precursor selection (SPS) option to maximize TMT quantitation sensitivity. Centroided data were collected for all MS3 scans.

Liver proteomic resultant data files were processed using Proteome Discoverer 2.1 (Thermo Fisher Scientific). MS2 data were queried against the Uniprot Mouse database using the Sequest algorithm [16]. A decoy search was also conducted with sequences in reversed order [17]. Data were filtered to a 1% peptide and protein level false discovery rate using the target-decoy strategy [17]. Reporter ion signal to noise values were used for quantitation. Spectra were used if the average signal to noise was greater than 10 across samples and if isolation interference was less than 25%. Data were normalized in a two-step process, whereby they were first normalized to the mean for each protein. To account for variation in the amount of protein labeled, values were then normalized to the median of the entire dataset. Final values are reported as normalized summed signal to noise per protein per sample. The unprocessed proteomics dataset is deposited in ProteomeXchange under the identifier PXD019618.

### Gene ontology and KEGG pathway enrichment analysis

**D**atabase for **A**nnotation, **V**isualization, and **I**ntegrated **D**iscovery (DAVID v6.8) [18] was used to perform Gene ontology (GO) and Kyoto Encyclopedia of Genes and Genomes (KEGG) pathway enrichment analysis with default settings on differentially expressed genes. GO biological processes and KEGG pathways with a P-value <0.05 and a gene count >2 were chosen as enriched. The top 5 enriched biological processes are represented as Chord plot using GOplot R package [19].

### Identification and comparison of human orthologs

Human orthologs of mouse genes were obtained using a “mouse to human” ortholog table downloaded from Ensembl through Biomart (www.ensembl.org/biomart/martview). The human orthologs of Differentially Expressed Genes (DEGs) were compared using Venny tool (http://bioinfogp.cnb.csic.es/tools/venny/).

### TCGA data analysis

From the Cancer Genome Atlas (TCGA) database, within the liver hepatocellular carcinoma (LIHC) dataset, level 3 RNA-seq data (including raw_read_count and scaled_estimate for each sample) corresponding to hepatocellular carcinoma (HCC, n = 361) and healthy liver (n = 50) were downloaded using the TCGA-assembler [20]. The Transcript per Million (TPM) values (obtained for each gene by multiplying scaled_estimate by 1, 000, 000) of HCC and normal samples were compared and statistical significance was obtained using unpaired student’s T-test. For the TCGA data, we utilized the Genomic Data Commons (GDC) data portal (https://portal.gdc.cancer.gov/) and UALCAN (http://ualcan.path.uab.edu/). The expression pattern of genes based on tumor histology is available in UALCAN (http://ualcan.path.uab.edu/cgi-bin/TCGAExResultNew2.pl?genenam=S100P&ctype=LIHC).

Univariate survival analysis was performed to assess the effect of gene expression level on HCC patient survival. HCC patients were categorized into two groups before the analysis, a) ***High expression***: patients with gene expression value greater than or equal to upper quartile (Q3) of total values and b) ***Low/Medium expression***: patients with gene expression value less than upper quartile (Q3) of total values. R packages such as “survminer” [http://www.sthda.com/english/rpkgs/survminer/] and “survival” [https://github.com/therneau/survival] were used to carry out analyses and to generate Kaplan Meir plots.

## Results

### Transcriptomic profiling reveals a distinct mRNA profile in the liver tissue of T5KO-HB mice susceptible to hepatocellular carcinoma

**Fig 1:**
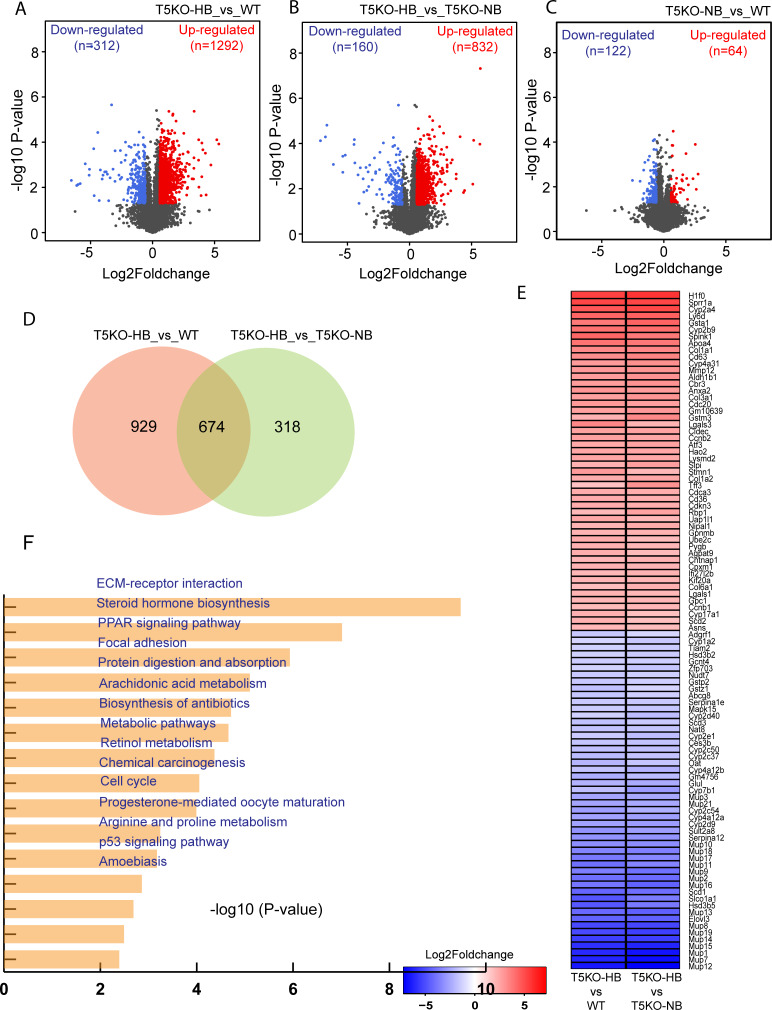
RNA-sequencing analysis of T5KO-HB, T5KO-NB and WT mice liver samples. (**A-C**) volcano plots showing distribution of differentially expressed genes (DEGs) in various comparisons. (**D**) Venn diagram showing statistics of genes differentially expressed in T5KO-HB samples compared to both WT and T5KO-NB samples. (**E**) Heatmap depicting top commonly DEGs in T5KO-HB samples. (**F**) Top 15 enriched KEGG pathways for common DEGs in T5KO-HB samples.

We previously described that a subset of Toll-like receptor 5 (TLR5)-deficient mice, which developed early onset hyperbilirubinemia, were prone to hepatocellular carcinoma (HCC) within 6 months of being fed an inulin-containing diet (ICD) [13]. Hepatic RNA sequencing analysis was performed in biological triplicates for the following experimental mouse groups fed inulin for 6 months: (i) wild type mice [WT] (n = 3), (ii) TLR5-deficient mice with normal bilirubin [T5KO-NB] (n = 3) and (iii) TLR5-deficient mice with high bilirubin [T5KO-HB] (n = 3). Differentially expressed genes (DEGs) were identified with an absolute fold change of 1.5 or more and a p-value <0.05. We observed an up-regulation of 1292 protein-coding genes and a down-regulation of 312 protein-coding genes in T5KO-HB mice compared to WT mice (**Fig 1A**). Additionally, 832 and 160 protein coding genes were up-regulated and down-regulated, respectively, in T5KO-HB mice when compared to T5KO-NB mice (**Fig 1B**). The comparison of T5KO-NB and WT mice showed only 186 DEGs (64 up-regulated and 122 down-regulated) in T5KO-NB compared to WT mice (**Fig 1C**). Among the DEGs in the three groups, 674 were found to be up-/down-regulated in T5KO-HB compared to WT and T5KO-NB mice (**Fig 1D**). The top 100 DEGs (50 up-regulated and 50 down-regulated) in T5KO-HB mice are shown in **Fig 1E**. Top 5 KEGG enriched pathways in T5KO-HB associated genes include ECM-receptor interaction, steroid hormone biosynthesis, PPAR signaling pathway, focal adhesion and protein digestion and absorption (**Fig 1F**). Furthermore, the top 5 biological enriched processes included mitotic nuclear division, cell cycle, cell division, negative regulation of lipid biosynthetic process and positive regulation of glucose metabolic process (**S1 Fig**).

### High throughput proteomic analysis reveals differentially expressed proteins in inulin fed HCC-prone T5KO-HB mice

**Fig 2:**
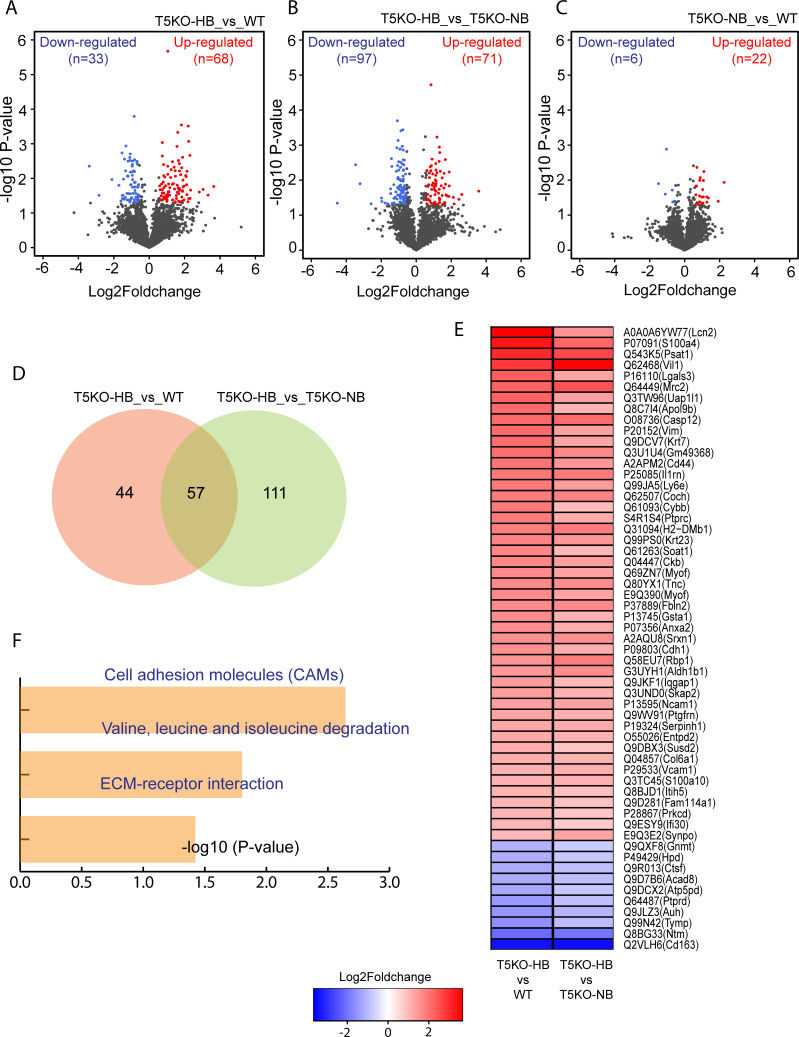
Proteomics data analysis of T5KO-HB, T5KO-NB and WT mice liver samples. (**A-C**) volcano plots showing distribution of differentially expressed proteins (DEPs) in various comparisons. (**D**) Venn diagram showing statistics of proteins differentially expressed in T5KO-HB samples compared to both WT and T5KO-NB samples. (**E**) Heatmap depicting top common DEPs in T5KO-HB samples. (**F**) Enriched KEGG pathways for common DEPs in T5KO-HB samples.

Tandem mass tag (TMT) based quantitative, multiplexed proteomic analysis was conducted using liver samples from inulin fed wild type mice [WT] (n = 3), TLR5-deficient mice with normal bilirubin [T5KO-NB] (n = 3) and TLR5-deficient mice with high bilirubin [T5KO-HB] (n = 4). Analysis showed 68 up-regulated and 33 down-regulated proteins in T5KO-HB compared to WT mice (**Fig 2A). Similarly**, 71 up-regulated and 97 down-regulated proteins were found in T5KO-HB compared to T5KO-NB mice (**Fig 2B**). Only 28 proteins (22 up-regulated and 6 down-regulated) showed altered expression in T5KO-NB compared to WT mice (**Fig 2C**). In total, 57 proteins were commonly altered in T5KO-HB compared to both T5KO-NB and WT (**Fig 2D**). **Fig 2E** lists all commonly altered proteins (47 upregulated and 10 downregulated) in T5KO-HB mice compared to WT and T5KO-NB mice. Cell adhesion molecules, valine, leucine and isoleucine degradation and ECM-receptor interaction enriched KEGG pathways were associated with the commonly altered proteins in T5KO-HB mice (**Fig 2F**). Top 5 biological enriched processes included cell adhesion, oxidation-reduction process, response to angiotensin, membrane raft assembly and negative regulation of cytokine-mediated signaling pathway (**S2 Fig**).

### T5KO-HB hepatic mouse genes are associated with human hepatocellular carcinoma and mortality

**Fig 3:**
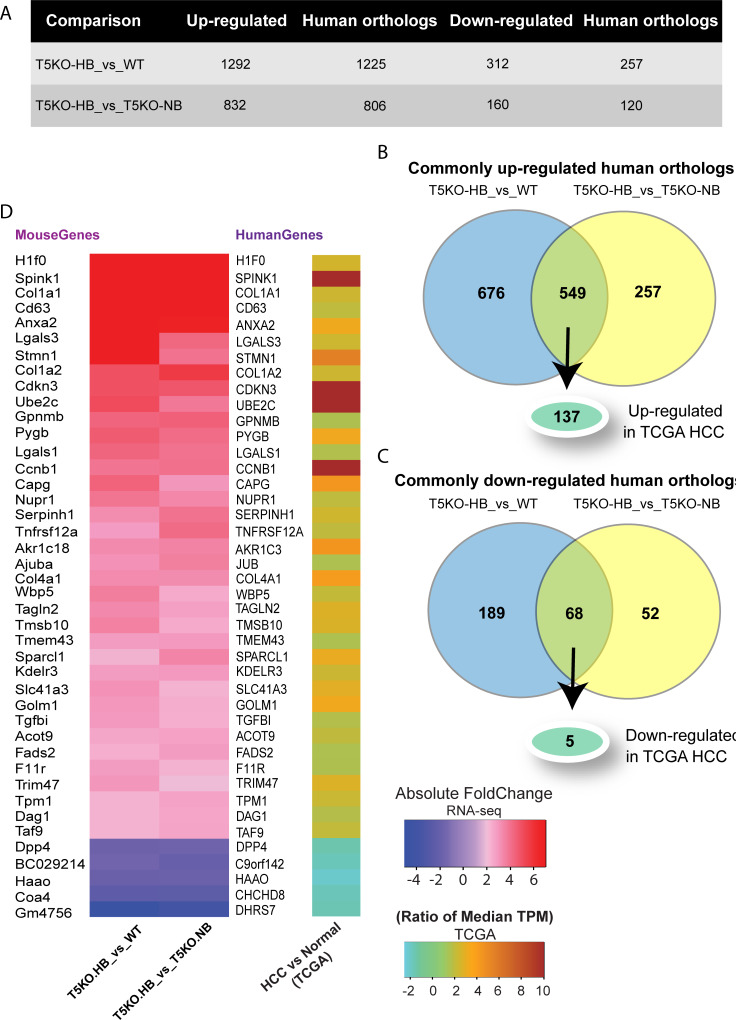
Comparative analysis of common T5KO-HB associated DEGs with TCGA human hepatocellular carcinoma dataset. (**A**) Statistics of human orthologs of differentially expressed genes (DEGs) in T5KO-HB compared to T5KO-NB and WT mice. Human orthologs obtained using Ensembl BioMart’s “mouse to human” ortholog table. (**B, C**) Comparison of up-regulated and down-regulated human orthologs and identifying HCC associated genes. (**D**) Heatmap listing top HCC associated genes.

In order to determine the role of T5KO-HB associated genes in hepatocellular carcinoma (HCC), we employed *in silico* gene expression and survival analyses in the Liver Hepatocellular Carcinoma (LIHC) dataset found in the Cancer Genome Atlas (TCGA) database. In the first step, we obtained the human orthologs of T5KO-HB associated mouse genes. Accordingly, we found that the human orthologous genes for 1225 out of 1292 mouse genes were up-regulated in T5KO-HB compared to WT mice, whereas 806 out of 832 mouse genes were up-regulated in T5KO-HB compared to T5KO-NB (**Fig 3A)**. Comparison between them showed 549 genes commonly up-regulated in T5KO-HB **(Fig 3B)**. Similarly, human orthologous genes were identified for 257 out of 312 mouse genes down-regulated in T5KO-HB compared to WT mice and 120 out of 160 mouse genes down-regulated in T5KO-HB compared to T5KO-NB mice (**Fig 3A**). Only 68 genes were common between the two sets of human orthologs (**Fig 3C**). **Fig 3D** shows the top over-/under-expressed specific human orthologous genes.

Next, we examined the mRNA expression pattern of T5KO-HB associated genes in HCC using the TCGA LIHC dataset comprising HCC (n = 361) and healthy liver (n = 50) samples. Expression pattern analysis of 549 up-regulated and 68 down-regulated human orthologs identified 142 genes with HCC specific expression. The 142 genes included 137 significantly over-expressed and 5 significantly under-expressed genes in HCC compared to normal (**Fig 3B–3D**). We performed Kaplan-Meier survival analysis of the 142 HCC specific genes to narrow down critical genes. We found association of 27 over-expressed genes with poor HCC patient survival (**Table 1**), while under-expressed genes showed no impact on patient survival. Among T5KO-HB associated genes that are over-expressed in human HCC and correlated with poor patient survival, ATIC, RUVBL1, ANXA2, BZW2 and TAGLN2 are involved in cell-cell adhesion, and RUVBL1, CCNB1 and DYNLT1 are involved in cell division.

**Table 1:** Statistics from the Kaplan Meir survival analysis of HCC specific genes leading to poor patient survival.

Gene	Log Rank P-value	High expression			Low/Median expression		
		# of Patients	# of deaths	Median survival (in days)	# of Patients	# of deaths	Median survival (in days)
**GARS**	1.62E-04	90	28	837	270	60	2131
**RUVBL1**	4.68E-04	91	25	1135	269	63	2116
**TAGLN2**	5.01E-03	90	28	1271	270	60	2131
**AKR1C3**	5.52E-03	91	26	1149	269	62	2116
**TAF9**	5.99E-03	91	25	1135	269	63	2116
**ATIC**	6.92E-03	91	25	1149	269	63	2131
**SAE1**	7.77E-03	91	25	1135	269	63	2131
**SERF1A**	9.37E-03	91	25	1135	269	63	2131
**SPINK1**	9.74E-03	90	28	1229	270	60	2131
**APRT**	1.00E-02	90	30	1229	270	58	2131
**CD63**	1.01E-02	90	31	1423	270	57	2116
**NOP58**	1.24E-02	90	22	899	270	66	2116
**SLC48A1**	1.26E-02	91	29	1147	269	59	2116
**ALDH18A1**	1.56E-02	91	24	1149	269	64	2116
**C3orf26**	1.67E-02	91	22	1490	269	66	2116
**CCNB1**	1.87E-02	91	26	1135	269	62	2116
**DYNLT1**	2.39E-02	90	25	1149	270	63	2131
**LAMB1**	2.70E-02	91	27	1149	269	61	2116
**ODC1**	3.74E-02	91	24	1372	269	64	2131
**CHCHD8**	3.76E-02	90	24	899	270	64	2116
**LGALS1**	3.80E-02	91	32	1490	269	56	2131
**GPNMB**	4.10E-02	90	31	1372	270	57	2116
**ACOT9**	4.50E-02	90	28	1149	270	60	2116
**BZW2**	4.60E-02	91	21	1490	269	67	2116
**PGD**	4.95E-02	91	26	1372	269	62	2116
**ANXA2**	4.98E-02	91	26	1372	269	62	2131
**PYGB**	4.98E-02	90	27	1372	270	61	2456

### PPAR associated genes are dysregulated in inulin-induced murine HCC and human hepatocarcinogenesis

**Fig 4:**
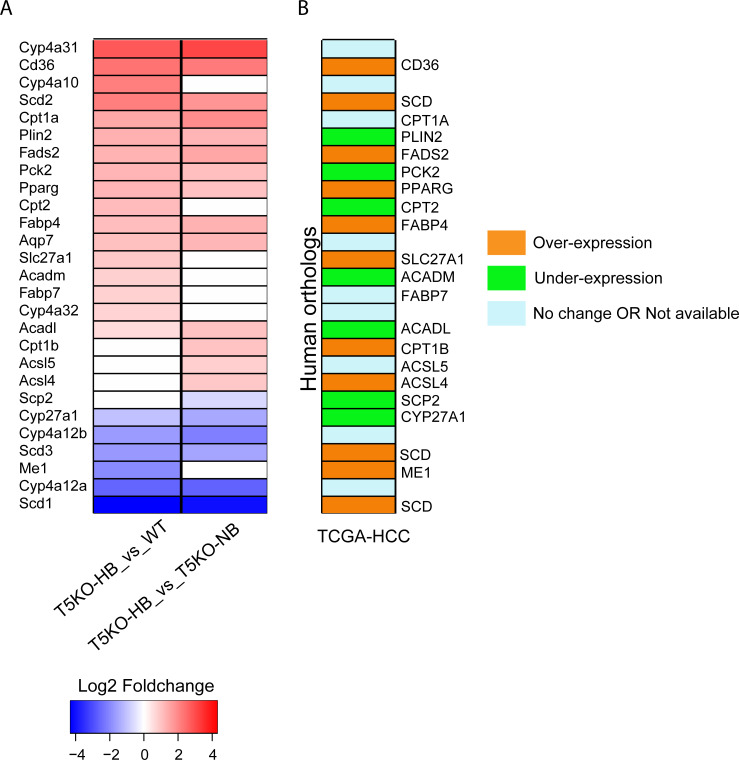
Comparative analysis of PPAR-associated DEGs in ICD-induced HCC with TCGA human hepatocellular carcinoma dataset. (**A**) Heatmap depicts top commonly PPAR-associated differentially expressed genes (DEGs) in T5KO-HB compared to T5KO-NB and WT mice. (**B**) After obtaining the human orthologs obtained using Ensembl BioMart’s “mouse to human” ortholog table, a comparison was conducted of up-regulated and down-regulated human orthologs of PPAR associated genes, which is depicted via a heatmap.

In our previous study, we highlighted that ICD-induced HCC was beneficially compensated with the alleviation of metabolic syndrome that is naturally prone in T5KO mice [13]. Accordingly, we have shown in the current study that 2 of the top 5 biological enriched processes in T5KO-HB mice are negative regulation of lipid biosynthetic process and positive regulation of glucose metabolic process (**S1 Fig**). Since both lipid and glucose metabolisms are strongly regulated by the peroxisome proliferator-activated nuclear receptor (PPAR) family, we next analyzed for the expression pattern of genes involved in PPAR signaling. Out of the 88 genes found to be involved in the PPAR pathway (KEGG ID: mmu03320), there were 27 DEGs (20 upregulated and 7 downregulated) between T5KO-HB mice and their controls (**Fig 4A**). We further expanded to determine whether these mouse DEGs in PPAR-mediated fatty acid metabolism and lipogenesis reflected human HCC. Accordingly, we observed 16 PPAR associated DEGs in human HCC, with 9 upregulated and 7 downregulated (**Fig 4B**).

## Discussion

The field of bioinformatics has become a pipeline to obtain high-throughput data and computational insights on a massive scale. This advancement in technology has introduced novel ‘omic’ fields such as genomics, proteomics, metabolomics, and lipidomics, which are collectively building the bridge to understand the link between genotype and phenotype [21]. Notwithstanding, these applications can be utilized to discover specific and sensitive biomarkers for diseases such as inflammatory bowel disease [22], cardiovascular diseases [23], liver diseases [24], and cancer [25, 26]. Uncovering these biomarkers would provide risk assessment of disease susceptibility, knowledge on disease progression, and clinical therapeutic targets. Another advantage to computational screening is the ability to ascertain human relevance of animal models that attempt to recapitulate human disease. In general, the laboratory mouse is widely considered a suitable organism for studying human relevant diseases because their gene expression patterns can recapitulate human conditions [11, 12]. To simulate human pathologies, the most common approaches include creating genetically engineered mouse models, introducing an exogenous substance (i.e. chemical, viral) to the animal, or performing a surgical procedure that triggers disease pathogenesis [27]. As expected, the human relevance of certain methods like chemical induction is often questioned as those substances may have low probability of being exposed to an individual. In the same manner, the practical usage of surgical methods could be questioned based on the high risk of post-surgery mortality.

Regardless of their limitations, chemical induction, viral infection, surgery, and genetic manipulation are the front-line approaches to study hepatocellular carcinoma (HCC) in a murine model [8–10]. HCC the is most frequent malignancy of the liver and is the fourth most common cause of cancer mortality worldwide [1]. Since HCC is a complex disease with a variety of etiological risk factors, bioinformatics can serve as an important tool to delineate the similarities and differences between HCC murine models and human hepatocarcinogenesis. Accordingly, there have been ‘omic’ studies that have explored gene expression profiles in distinct mouse models of HCC and compared to human HCC biology. Dow et al. [28] utilized genomics and transcriptomic profiles to determine human relevance of four different etiological models of murine HCC: the Stelic Animal Model (STAM), liver-specific TAK-1 deficient mice, MUP transgenic murine model, and carcinogen-driven diethylnitrosamine (DEN)-induced HCC. Intriguingly, tumors from STAM mice were more molecularly similar to human HCC, whereas TAK1 tumors exhibited a comparative mutational signature with low-grade human HCC tumors [28]. Out of the models, tumors collected from DEN-treated mice had the least similarity to human HCC, which was associated with a *Braf* V637E genetic mutation that is rarely found in humans [28]. Considering that cirrhosis is a pre-neoplastic indicator of HCC susceptibility, the fact that the DEN model of HCC is cirrhosis-independent [29] might explain why it has the least similarity to human HCC. Comparatively, methionine adenosyltransferase 1A-deficient mice that are prone to HCC were found to have differentially expressed genes in one carbon, glucose and fatty acid metabolism that were analogous to human HCC and cirrhotic livers [30]. While the mentioned murine HCC models do present merits, these studies still emphasize that novel murine HCC models are still warranted to recapitulate human HCC and that bioinformatics may be the tool to identify and confirm human relevance.

We undertook this study to determine whether our recent discovery of a dietary fermentable fiber-induced HCC murine model could have similar gene and protein profiles to human HCC. The first step involved determining differentially expressed genes (DEGs) in HCC mice compared to their relative control. Accordingly, transcriptome profiling revealed that there were 674 DEGs in HCC mice compared to their healthy control. Many of the upregulated DEGs in the mice that developed dietary fermentable fiber-induced HCC included both known tumor-associated genes along with tumor suppressors. For example, the most upregulated gene was *H1f0*, which is known to have dynamic epigenetic influence and has been associated to sustain long-term cancer growth [31]. Additionally, there were multiple collagen genes overly-expressed in HCC mice along with *Ly6D*, *Tff3*, and *Spink1*, which are well known HCC markers [32–35]. Alongside, *Gsta1* and *Atf3* were upregulated in HCC mice, which have been proposed as potential tumor suppressors in HCC development [36–39]. Intriguingly, there were 17 genes associated with the group of major urinary proteins (MUP) that were downregulated in the T5KO-HB mice prone to ICD-induced HCC. MUP family members are known sensors and regulators of nutrient metabolism, where defects in this system has been thought to contribute to metabolic diseases [40]. It is noteworthy that previous reports have associated a decrease in MUP to be indicative of early liver tumor development [41, 42]. It is further interesting to acknowledge that MUP-1 in germ-free mice has been found to be significantly decreased compared to conventional, specific pathogen free mice [43], which suggests that the expression of MUP-1 and other MUP family members may be regulated by the gut microbiota. Considering that ICD-induced HCC is both nutrient and gut microbiota-dependent, the results from this study could indicate MUP as a therapeutic target for function restoration.

Kyoto Encyclopedia of Genes and Genomes (KEGG) pathway analysis uncovered enrichment in extracellular matrix (ECM)-receptor interaction, steroid hormone biosynthesis, PPAR signaling pathway, focal adhesion and protein digestion and absorption during ICD-induced HCC. Additionally, the top 5 biological enriched processes included mitotic nuclear division, cell cycle, cell division, negative regulation of lipid biosynthetic process and positive regulation of glucose metabolic process. When next analyzing for PPAR signaling associated genes, which reflects modulation of both lipid and glucose metabolisms, we observed 27 DEGs that are associated with hepatic steatosis, steatohepatitis and hepatocarcinogenesis. To highlight a few, T5KO-HB mice had a severe downregulation of stearoyl-CoA desaturase 1 (SCD1), which could characterize an increased susceptibility toward hepatic fibrosis but less steatosis development when the tumorigenesis dominates the liver [44]. SCD1 activity and *de novo* lipogenesis has been regarded to be necessary for HCC progression [45]. Contradictory, there is evidence suggesting that SCD1 is dispensable for *de novo* lipogenesis and hepatic carcinoma, whereas SCD2 was strongly upregulated during liver cancer progression [46]. Excitingly, our ICD-induced HCC model indicated a significant upregulation of SCD2, along with other lipogenic and HCC associated genes such as CD36 and PPARγ [47, 48]. When further analyzing by tandem mass tag based quantitative, multiplexed proteomic analysis, we demonstrated that the over-expressed proteins were associated with cell adhesion molecules, valine, leucine and isoleucine degradation and ECM-receptor interaction. These results are analogous to previous reports that have observed common DEPs tightly associated with the cell cycle, ECM-receptor interactions, as well as protein digestion and absorption pathways, that were shared between human and mouse HCC [49, 50].

The Cancer Genome Atlas (TCGA) is one of the most successful cancer genomics database to date [51]. After obtaining the human orthologs of the mouse genes, we did a comparison analysis to level 3 RNA-seq data found in the TCGA database, corresponding to human HCC and healthy liver samples. Out of the 549 up-regulated and 68 down-regulated human orthologs identified, 142 genes (137 significantly over-expressed and 5 significantly under-expressed) were associated with human HCC. *Spink1* and *UBE2C* were two of the highest upregulated human orthologs when comparing mouse to human HCC, both of which have been considered as potent HCC therapeutic targets [34, 52]. We observed that genes involved in cell-cell adhesion (ATIC, RUVBL1, ANXA2, BZW2 and TAGLN2) and cell division (RUVBL1, CCNB1 and DYNLT1) were significantly over-expressed in human HCC and associated with poor patient survival. Both ATIC and BZW2 are considered human oncogenic genes that promote cancer proliferation and migration through mTOR signaling [53, 54]. Comparatively, TAGLN2 is known as a tumor suppressor whose protective effects are inhibited when phosphorylated by a cdc2-related serine/threonine protein kinase [55]. Alongside, we found that both PPAR-associated fatty acid metabolism associated genes, CD36 and PPARγ, were significantly upregulated in human HCC patients. Hence, these genes might serve as molecular targets to restrict oncogenic (i.e. ATIC, BZW2) and lipogenic (i.e. CD36, PPARγ), but promote tumor suppressive (i.e. TAGLN2) activity to treat human HCC. Altogether, the mentioned signatures highlight potential underlying mechanisms in ICD-induced HCC that are related to human HCC.

While animal models are essential for the molecular characterization of any human disease/disorder, they do present certain limitations and our model is no exception. As mentioned earlier, human cancers including HCC have highly variable etiologies. In our HCC model, liver cancer progression is diet- and microbiota-dependent, which could be considered as a non-conventional approach to study HCC when compared to viral or chemical induction methods. Additionally, the early onset of hyperbilirubinemia and cholemia depicts that ICD induces a cholestatic sub-type of HCC, which limits to a specific etiology of liver cancer development. Furthermore, our model utilized Toll-like receptor 5 deficient (T5KO) mice that have a compromised innate immune system, which we have previously described increased their susceptibility to metabolic syndrome [56]. Despite these potential drawbacks, we believe that robust fermentable fiber-induced HCC offers as a promising model for the following reasons: (i) presents a novel approach to study metabolic function to tumor formation, analogous to HCC susceptibility found in Acox-deficient mice that have impaired fatty acyl-CoA oxidase and β-oxidation [57], (ii) serves as an alternative model to study cholestatic HCC compared to previous mouse models with genetic ablation of either ABCB4 or MDR2 [58], (iii) allows to mechanistically understand the role of the gut microbiota in HCC, which is becoming well recognized as a strong influencer through the gut-liver axis [59], and (iv) this model recapitulates known consequences of cholestasis that is exhibited in humans such as depletion of essential fatty acids and fat-soluble vitamins [60]. Notwithstanding, HCC incidence has been continuing to increase for the past couple of decades, and while this is reported to be largely due to viral infection, refined fermentable fibers may be another unknown risk factor. Our model may fulfil the requirement to understand the molecular underpinnings of fermentable fibers in cholestasis and HCC, which is severely under-explored. Overall, this study supports the ICD-induced HCC murine model as human relevant and instigates its capability to be utilized for future translational studies in understanding human HCC pathogenesis along with determining clinical molecular targets.

## Conclusion

Altogether, our integrative transcriptomic and proteomic study suggests that ICD-induced HCC in mice recapitulates gene signatures found in human liver cancer, which highlights potential underlying mechanisms related to human HCC. This study further instigates the human relevance of the ICD-induced HCC murine model, which can be utilized for future translational studies to better understand human HCC pathogenesis and to determine molecular therapeutic targets.

## Supporting information

S1 Fig Enrichment of GO Biological processes using DEGs in T5KO-HB obtained from RNA-seq. Chord plot displaying relationship between top 5 GO biological processes and DEGs. Association between gene and biological process is indicated via ribbons and DEGs are arranged based on log2Foldchange.
(EPS)

S2 Fig Enrichment of GO Biological processes using DEGs in T5KO-HB obtained from proteomics data. Chord plot displaying relationship between top 5 GO biological processes and DEGs. Association between gene and biological process is indicated via ribbons and DEGs are arranged based on log2Foldchange.
(EPS)
